# OUTCOMES OF A PILOT PRAGMATIC TRIAL IMPLEMENTING PALLIATIVE CARE IN NURSING HOME CARE

**DOI:** 10.1093/geroni/igad104.1366

**Published:** 2023-12-21

**Authors:** Joan Carpenter, Laura Hanson, Nancy Hodgson, Amy Jackson, Merve Gurlu, Shijun Zhu, Mary Ersek

**Affiliations:** University of Maryland School of Nursing, Baltimore, Maryland, United States; University of North Carolina at Chapel Hill, School of Medicine, Chapel Hill, North Carolina, United States; University of Pennsylvania School of Nursing, Philadelphia, Pennsylvania, United States; University of Maryland, Baltimore, Maryland, United States; University of Maryland, Baltimore, Baltimore, Maryland, United States; University of Maryland School of Nursing, Baltimore, Maryland, United States; University of Pennsylvania, Philadelphia, Pennsylvania, United States

## Abstract

Palliative care that aligns treatments with preferences for people living with serious illness in nursing homes (NHs) can improve quality of life. Given limited access to specialty palliative care in NHs, enhanced primary palliative care is needed. We conducted a pilot pragmatic clinical trial to establish feasibility, acceptability, and preliminary effectiveness of an NH primary palliative care intervention. Nurse practitioners, employed by each intervention site, attended a 5-hour virtual course, one-day intensive workshop, and monthly 1-hour facilitation sessions to enhance palliative care practices. We recruited residents living with serious illness and palliative care needs from 12 NHs in the MidAtlantic US. Using a nonequivalent group design, participants were clustered by site and assigned to intervention or control group in a 2:1 (respectively) ratio. Our primary outcome was the Palliative Outcome Scale Version 2 (POSv2, range 0-40). Of 318 residents screened, 119 met inclusion criteria, and 45 consented for participation (n=35 intervention; n=10 control). Participants (mean age=86) were mostly White (98%), female (76%), and able to make their own decisions (58%). Clinician intervention fidelity and participant acceptability were high. At 21 days, quality of life did not differ between control (POSv2=8.79) and intervention groups (POSv2=10.03; p=0.72). We conclude that an NH primary palliative care intervention is feasible and acceptable; effectiveness to improve quality of life may depend upon the successful implementation of a highly tailored intervention and longer-term follow-up.

